# A New Method for Non-Destructive Identification and Tracking of Multi-Object Behaviors in Beef Cattle Based on Deep Learning

**DOI:** 10.3390/ani14172464

**Published:** 2024-08-24

**Authors:** Guangbo Li, Jiayong Sun, Manyu Guan, Shuai Sun, Guolong Shi, Changjie Zhu

**Affiliations:** 1 College of Electronic and Information Engineering, Huaibei Institute of Technology, Huaibei 235000, China; liguangbo@hblgxy.edu.cn (G.L.);; 2 School of Economics and Management, Huaibei Institute of Technology, Huaibei 235000, China; 3 School of Information and Artificial Intelligence, Anhui Agricultural University, Hefei 230036, China

**Keywords:** beef cattle behavior identification, YOLOv8, Deep SORT, multi-object tracking, re-identification network

## Abstract

**Simple Summary:**

Through the non-destructive recognition and tracking algorithm of multi-objective behaviors of beef cattle, practitioners are able to obtain beef cattle feeding information in an all-round way, which lays the foundation for intelligent farming. This study is based on the deep learning recognition algorithm YOLOv8 and tracking algorithm Deep SORT. Through optimizing the convolution module, introducing the attention mechanism, improving the re-identification network, and the trajectory generation and matching process, a new method for the non-destructive identification and tracking of multi-target behaviors of beef cattle is constructed. The average accuracy of nine behaviors can be up to 96.5%, and the accuracy of multi-target tracking is up to 92.1%, which can provide technical support for beef cattle management.

**Abstract:**

The method proposed in this paper provides theoretical and practical support for the intelligent recognition and management of beef cattle. Accurate identification and tracking of beef cattle behaviors are essential components of beef cattle production management. Traditional beef cattle identification and tracking methods are time-consuming and labor-intensive, which hinders precise cattle farming. This paper utilizes deep learning algorithms to achieve the identification and tracking of multi-object behaviors in beef cattle, as follows: (1) The beef cattle behavior detection module is based on the YOLOv8n algorithm. Initially, a dynamic snake convolution module is introduced to enhance the ability to extract key features of beef cattle behaviors and expand the model’s receptive field. Subsequently, the BiFormer attention mechanism is incorporated to integrate high-level and low-level feature information, dynamically and sparsely learning the behavioral features of beef cattle. The improved YOLOv8n_BiF_DSC algorithm achieves an identification accuracy of 93.6% for nine behaviors, including standing, lying, mounting, fighting, licking, eating, drinking, working, and searching, with average 50 and 50:95 precisions of 96.5% and 71.5%, showing an improvement of 5.3%, 5.2%, and 7.1% over the original YOLOv8n. (2) The beef cattle multi-object tracking module is based on the Deep SORT algorithm. Initially, the detector is replaced with YOLOv8n_BiF_DSC to enhance detection accuracy. Subsequently, the re-identification network model is switched to ResNet18 to enhance the tracking algorithm’s capability to gather appearance information. Finally, the trajectory generation and matching process of the Deep SORT algorithm is optimized with secondary IOU matching to reduce ID mismatching errors during tracking. Experimentation with five different complexity levels of test video sequences shows improvements in IDF1, IDS, MOTA, and MOTP, among other metrics, with IDS reduced by 65.8% and MOTA increased by 2%. These enhancements address issues of tracking omission and misidentification in sparse and long-range dense environments, thereby facilitating better tracking of group-raised beef cattle and laying a foundation for intelligent detection and tracking in beef cattle farming.

## 1. Introduction

With the accelerated process of urbanization and the increase in people’s income levels, the demand for meat in the market continues to rise [1]. This has led to a rapid increase in the demand for livestock, represented by beef cattle, for which people have higher requirements regarding welfare and production efficiency. As a pillar industry in China’s animal husbandry, the beef cattle farming industry aims to assess the welfare status and behavioral conditions of beef cattle more effectively. Real-time monitoring and behavior tracking of beef cattle are essential for feedlot cattle farming to promptly detect any anomalies in the cattle. Once anomalies are detected, timely human intervention can be carried out to reduce losses and effectively enhance the profitability of large-scale beef cattle farming [2,3].

Traditional manual monitoring and identification methods suffer from high costs and low efficiency. In wearable sensor identification, which to some extent reflects the behavior of beef cattle, the extensive use of sensors may influence the behavior of the cattle. This can lead to misunderstood abnormal activities during the decision-making process, thereby affecting the tracking effectiveness. Furthermore, wearable sensors incur high material costs and have limited battery life, which could potentially impact the environment [4,5,6]. Therefore, the use of non-contact deep learning algorithms for intelligent detection and tracking of beef cattle has become a hot topic in beef cattle farming research. The work of domestic and foreign experts is as follows:

Zheng et al. [7] proposed a new method for beef cattle behavior identification and tracking based on the YOLOv5 detector and six tracking algorithms, including Deep SORT. This method first prunes the YOLOv5 detector model, then introduces cascaded buffering to further integrate the appearance features of detection with the motion features of trajectories. This achieves a multi-object tracking precision (MOTP) of 86.1% and an average data association ratio (IDF1) score of 80.3%. Zheng et al. [8] also introduced a multi-object tracking algorithm based on YOLO v7 and ByteTrack. This method enhances detection accuracy by adding self-attention and convolutional fusion modules on top of YOLO v7 and optimizing the spatial pyramid pooling section. The parameters of the improved ByteTrack algorithm were optimized for the Kalman filter to enhance tracking effectiveness. The multi-object tracking accuracy (MOTA) and average data association ratio (IDF1) reached 75.4% and 77.8%, respectively, showing a 6.1% and 3.8% improvement over the original version, with a 37.5% reduction in ID switch times (IDS). Bhujel et al. [9] proposed a deep learning-based pig behavior and motion activity detection and tracking algorithm, comparing the detection accuracy of the Faster R-CNN and YOLOv4 detectors. They found that the combination of the YOLOv4 detector with the Deep SORT tracking algorithm performs better in pig positioning and detection, thus being suitable for multi-target detection and tracking in pigs. Tassinari et al. [10] identified individual beef cows and tracked them by optimizing the YOLO v3 detector. Myat Noe et al. [11] proposed new methods for black cattle identification using the YOLO v5 and YOLO v7 algorithms and the improved Deep SORT algorithm for multi-target tracking of black cattle. T. Psota E et al. [12] proposed a probabilistic detection-based tracking method that utilizes a detector to detect the key points of each animal as inputs, immediately followed by the use of a classification network to assign unique identifiers to the instances and an algorithm to assign ear marking aliasing probabilities, and finally the use of a fixed cardinality of the target to create a set of consecutive trajectories and real-time tracking in hardware.

In conclusion, compared to traditional beef cattle tracking methods, deep learning-based multi-object tracking algorithms offer advantages such as high tracking accuracy and fast data processing. However, there is still room for improvement. (1) Due to the variety in beef cattle behaviors and the high similarity of some behaviors, there is room for improvement in the detector’s accuracy in identifying cattle behaviors. (2) In complex scenarios with different lighting and density levels, the re-identification network needs improvement in acquiring beef cattle behavioral appearance information. (3) ID mismatches and misalignments still account for a significant proportion of beef cattle tracking algorithms.

Based on the aforementioned issues, the research objectives of this study are outlined as follows:

(1) Theoretical objective: On the basis of the Deep SORT tracking algorithm, replace the detector with YOLOv8n_BiF_DSC to enhance detection accuracy, then replace the re-identification network model to improve the tracking algorithm’s appearance information acquisition capability. Finally, optimize the trajectory generation and matching process of the Deep SORT algorithm with secondary IOU matching to reduce ID mismatches and misalignments during tracking.
(2) Practical objective: Enhance the accuracy of beef cattle behavior identification and tracking in complex scenarios with different lighting and density levels through dataset enhancement and algorithm optimization. The aim is to provide technical support for subsequent applications such as beef cattle behavior early warning, intelligent farming, and management.

## 2. Materials and Methods

### 2.1. Dataset

**Figure 1:**
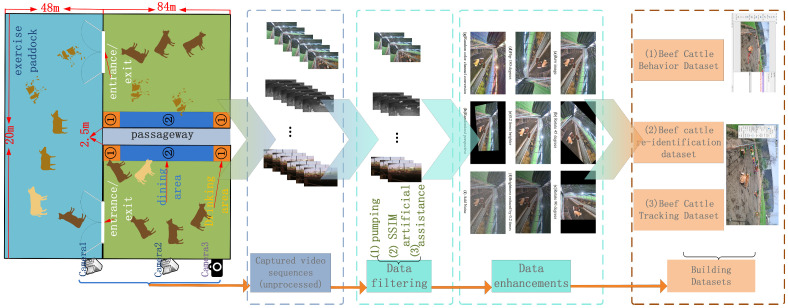
Data collection and dataset construction process.

In order to better achieve beef cattle behavior identification and tracking, the dataset in this study is divided into the following three stages: The first stage involves beef cattle data collection, providing raw data for beef cattle behavior identification and tracking. The second stage is data preprocessing. The third stage involves constructing the dataset, which includes a beef cattle behavior detection dataset, a beef cattle re-identification dataset, and a beef cattle tracking dataset. The process of data collection and dataset construction is illustrated in Figure 1.

#### 2.1.1. Collection of Dataset

The beef cattle data were collected at Shengtu Livestock Farm in Huaibei City, Anhui Province. The data collection equipment included fixed collection equipment (A LINE OF DEFENSE cameras) and mobile collection equipment (Huawei Mate20 Pro smartphones (Huawei, Shenzhen, China)). In order to obtain more comprehensive beef cattle behavior data, this study collected daily videos of beef cattle over 7 days in various scenarios (different lighting conditions, different levels of density, etc.). The video durations ranged from 1 min to 30 min, effectively reflecting the characteristics of beef cattle behavior, enriching the robustness of the beef cattle behavior, re-identification, and tracking datasets.

#### 2.1.2. Preprocessing of Beef Cattle Behavior Detection Dataset

**Figure 2:**
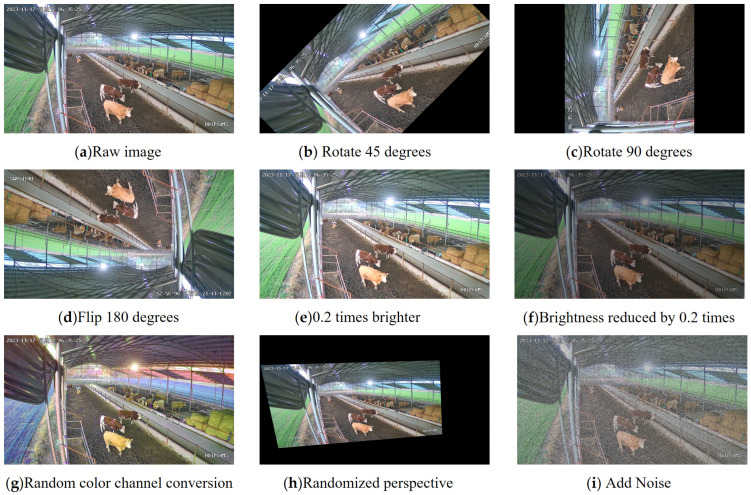
Data enhancement example.

The preprocessing process for the beef cattle behavior detection dataset is as follows:

(1) Data filtering: The collected videos were processed by extracting frames, with one image taken every 15 frames to reduce image similarity. Then, structural similarity index (SSIM [13]) filtering was applied to adjacent pairs of image frames to enhance data feature diversity. Finally, data samples that met the target dataset criteria were manually selected.
(2) Data augmentation: Eight types of data augmentation techniques were applied to the data samples, including clockwise rotation by 45 degrees and 90 degrees, flipping by 180 degrees, increasing and decreasing brightness by 0.2 times, random color channel transformation, random perspective transformation, adding noise, and other data augmentations. This expanded the experimental samples to eight times the original size, as shown in Figure 2. This process reflected the posture characteristics of beef cattle in complex scenes, providing ample data support for algorithm identification and tracking and enhancing the robustness of identification and tracking algorithms.

(3) Building a beef cattle behavior detection dataset: The beef cattle behaviors include standing, lying, mounting, fighting, licking, eating, drinking, working, and searching, totaling nine behaviors. The identification rules and instance information are shown in Table 1. After augmenting the data, 34,560 images of beef cattle behavior were annotated using labelImg (1.8.0) software, and the dataset was split into training and validation sets in an 8:2 ratio.

#### 2.1.3. Preprocessing of the Beef Cattle Re-Identification Dataset

**Figure 3:**
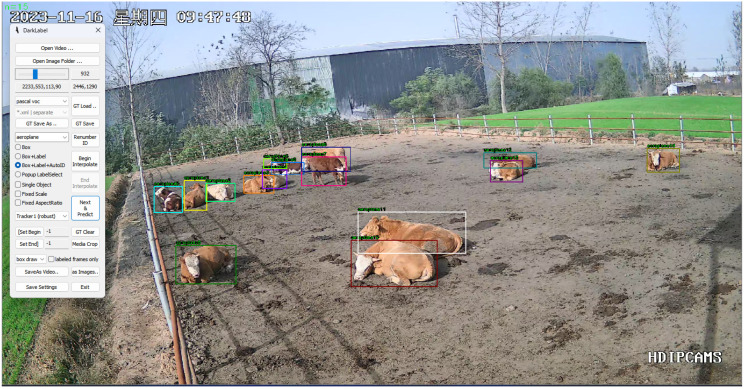
DarkLabel labeling interface. (“星期四” is Thursday).

In order to rapidly and accurately obtain a dataset for re-identification networks to provide appearance features for tracking algorithms, this study utilized the lightweight video annotation software Dark Label (2.3), as shown in Figure 3 (https://github.com/darkpgmr/DarkLabel/releases/download/darklabel2.3-update2/DarkLabel2.3-update2.zip, accessed on 20 August 2024).

**Figure 4:**
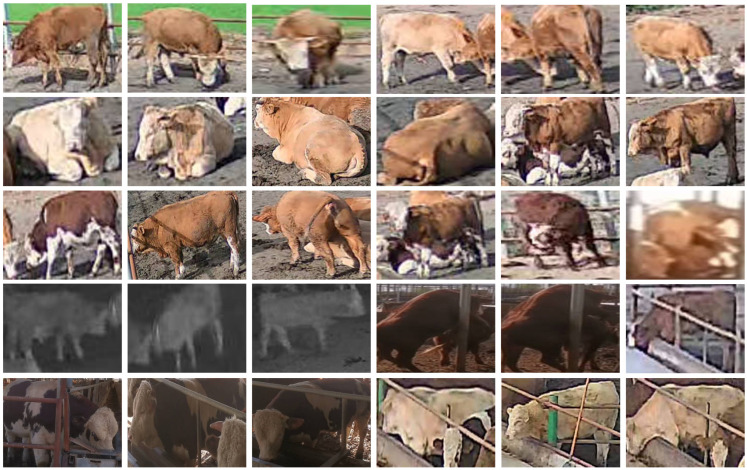
Sample appearance re-recognition dataset.

Due to the lack of publicly available beef cattle re-identification datasets, this research constructed its own beef cattle re-identification dataset. Multiple segments of beef cattle videos were collected for the re-identification network dataset, with video durations ranging from 1 to 3 min, each containing varying numbers of individual beef cattle. To enhance the accuracy of individual beef cattle re-identification, it is crucial to avoid the presence of the same beef cattle in different video sequences, ensuring the uniqueness of the individuals in the video sequences used. This necessitates manual filtering and integration of the collected video sequences. Subsequently, the effective video sequences were annotated frame by frame using the Dark Label software, where different labels in each frame represent different beef cattle individuals, thus indicating different individuals. Finally, these annotations were transformed into the Market-1501 dataset format through code scripts to establish the beef cattle re-identification dataset. The dataset consists of 45 beef cattle, totaling 3150 images, with an average of approximately 40 and 30 images for the training and validation sets per beef cattle individual, respectively. Examples from the beef cattle re-identification dataset are shown in Figure 4.

#### 2.1.4. Preprocessing of Beef Cattle Tracking Dataset

To validate the effectiveness of multi-target tracking of group-raised beef cattle in actual environments, this study selected 5 representative beef cattle videos of varying durations (1–3 min) for frame-by-frame annotation. The annotation process was carried out using the DarkLabel software, followed by conversion into the MOT-16 dataset format using code scripts specifically designed for tracking evaluation. Subsequently, the tracking performance was evaluated using the evaluation code for MOT-16 metrics to assess the accuracy of the tracking results.

### 2.2. Improved Method

**Figure 5:**
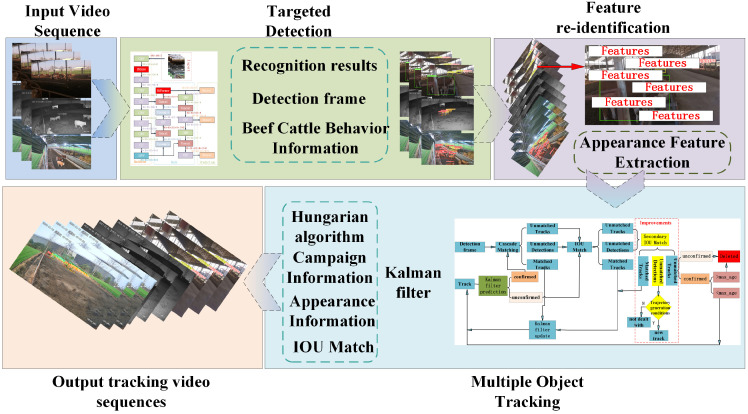
Framework for nondestructive identification and tracking of beef cattle behavior.

This paper proposes a new method for the non-destructive identification and tracking of beef cattle behaviors based on YOLOv8 and Deep SORT [14]. This method can detect beef cattle behaviors in real time and track cattle individuals, providing precise information for cattle husbandry practitioners. The overall framework of this method, as shown in Figure 5, consists of the following steps: Firstly, the improved YOLOv8 detector is used to detect the current frame, identifying the bounding box positions of the beef cattle and their behavior data. Next, feature extraction is performed on the current frame using a re-identification network to obtain appearance features. Finally, the detection results and feature extraction information are simultaneously input into the Deep SORT algorithm, which assigns IDs to cattle individuals through processes such as Kalman filtering, the Hungarian algorithm for object association, etc., enabling the non-destructive identification and tracking of beef cattle behaviors.

**Figure 6:**
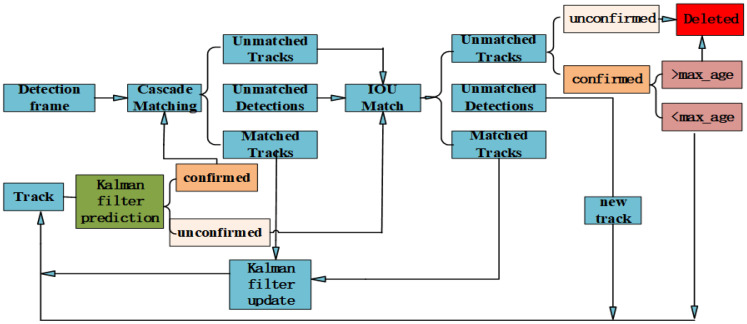
Deep SORT target tracking process.

The Deep SORT algorithm enhances the SORT [15] algorithm by incorporating cascaded matching and confirming new trajectories. Trajectories are categorized into two states: confirmed and tentative. New trajectories start as tentative and transition to confirmed if they match continuously for a threshold (the default is 3). Confirmed trajectories are deleted if they mismatch consecutively beyond a time threshold. The specific workflow of the Deep SORT algorithm is illustrated in Figure 6.

The entire algorithm is as follows:

(1) Create trajectories corresponding to the detections in the first frame, with trajectories in a tentative state, and predict the bounding boxes corresponding to the trajectories using Kalman filtering.
(2) Calculate the cost matrix by matching the detection boxes obtained from the current frame with the predicted boxes from the previous frame using intersection-over-union (IOU) matching.
(3) Apply the cost matrix from the previous step to the Hungarian algorithm to obtain three possible outcomes: trajectory mismatch, detection box mismatch, and trajectory match.
(4) Repeat the first two steps until a trajectory transitions into a confirmed state for the next step, or the last frame of the video is reached.
(5) Directly match the uncertain states predicted by Kalman filtering using IOU matching, while confirmed-state predicted boxes need to be cascaded and matched with detection boxes.
(6) Cascaded matching results in three possible outcomes: the first is updating the trajectory variables directly for trajectory matches, while the other two involve performing IOU matching for detection boxes and trajectory mismatches.
(7) Based on the results from the previous step, three possibilities arise: trajectory match, which follows the same process as the previous step; detection box match, which assigns a new trajectory; and trajectory mismatch, which can lead to two scenarios: if the trajectory is in a tentative state, it is deleted directly, whereas if it is in a confirmed state and the mismatch count exceeds a time threshold, it is deleted; otherwise, it is retained.
(8) Repeat steps 5 to 7 until completion.

#### 2.2.1. Detector Improvement

The target detection network used in the Deep SORT algorithm is the two-stage Faster R-CNN [16] algorithm. Although the Faster R-CNN algorithm has high accuracy, its algorithm parameters are immense, leading to slower detection speeds that do not adequately meet the requirements of multi-target tracking in group-raised beef cattle environments. To achieve more precise and faster multi-target tracking of beef cattle, this study introduces the one-stage [17,18,19,20] YOLOv8 algorithm and its improved version, YOLOv8n_BiF_DSC.

Developed by the Ultralytics team, YOLOv8 inherits the strengths of YOLOv5 and YOLOv7 [21] in its design and further enhances and optimizes them. YOLOv8 replaces the C3 module with the more feature-rich and reasonably channel-sized C2f module compared to YOLOv5. It optimizes the convolution operations after up-sampling in the PAN-FPN in the neck network and upgrades the prediction head to the decoupled head. YOLOv8 shows improvements in algorithm accuracy, computation time, and model size to varying degrees. YOLOv8 officially provides five versions: YOLOv8n, YOLOv8s, YOLOv8m, YOLOv8l, and YOLOv8x, where the sizes and detection accuracies increase sequentially while detection speeds decrease. Considering the practical needs of beef cattle farming scenarios, this study adopts the fastest detection speed and smallest model size, YOLOv8n, as the base model. To better meet the requirements of beef cattle posture identification in diverse scenarios, like varying lighting conditions and different levels of density, the following improvements were made: (1) Enhancing the efficiency of feature extraction in the backbone network by introducing the dynamic snake convolution module, which conforms to the algorithm structure for feature learning without deviating from the target structure; (2) introducing the BiFormer attention mechanism to enhance the correlation between global and local features, facilitating the fusion of high- and low-level information to enhance beef cattle target identification capabilities.

**Figure 7:**
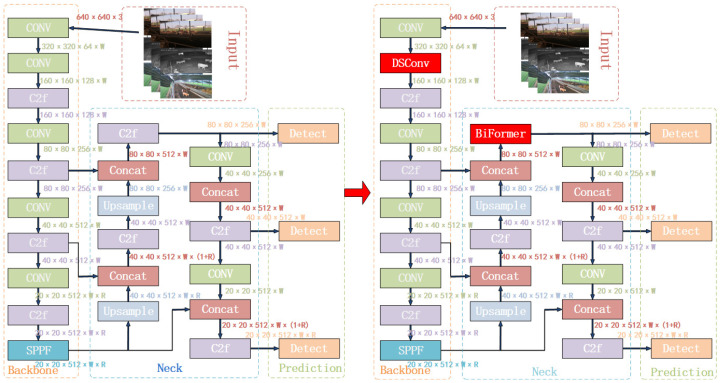
YOLOv8n_BiF_DSC algorithm flow.

The improved YOLOv8n_BiF_DSC algorithm model is illustrated in Figure 7. The specific improvements are outlined as follows:

##### Introduction to the Dynamic Snake Convolution (DSConv) Module

**Figure 8:**
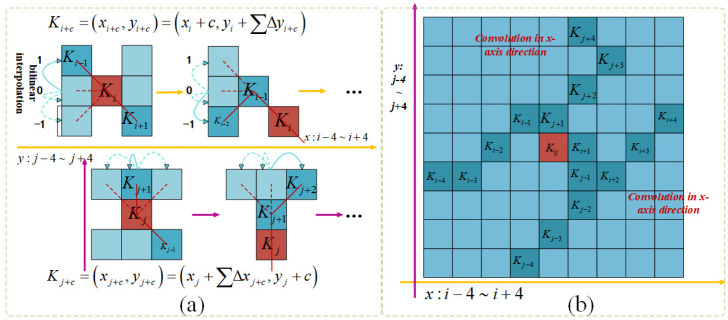
DSConv graphical representation. (**a**) DSConv coordinate calculation. (**b**) DSConv sensory field.

Given the limited pixel representation of beef cattle behavioral structures within images and the intricate environmental disturbances like varying lighting, density, and occlusions present in real-world cattle farming scenarios, the process of extracting model features becomes notably challenging, ultimately resulting in a decrease in detection accuracy. Therefore, the extraction of key features of beef cattle behavior plays a crucial role in the model’s identification performance. This paper introduces the Dynamic Snake Convolution (DSConv [22]) module to extract key features of beef cattle behavior. By changing the shape of the convolution kernel to better align with the dispersed features of beef cattle behavior, the module ensures that the essential features are not deviated from. The feature extraction process of Dynamic Snake Convolution (DSConv) is illustrated in Figure 8a.

Given the standard 2D convolution coordinates as *K*, with the center coordinates being *K_i_* = (*x_i_*, *y_i_*), a 3 × 3 convolution kernel *K* with a dilation rate of 1 is represented as follows:

(1) K={(x−1,y−1),(x,y−1),⋯,(x+1,y+1)}

∆ represents the deformation offset, which is beneficial for the convolution kernel to focus on the behavioral features of beef cattle. Straightening the standard convolution kernel in the *x*-axis and *y*-axis directions, the kernel size is 9. Taking the *x*-axis direction as an example, the positions of the grids in *K* are denoted as follows: *K*_*i*±*c*_ = (*x_i_* ± *c*, *y_i_* ± *c*), where *c* = {0, 1, 2, 3, 4} represents the horizontal distance from the central grid. Compared to *K_i_*, *K_i_* + 1 increases by an offset amount ∆ = {*δ*|*δ ε* [−1, 1]}. *K*_*i*±*c*_ in the *x*-axis direction is as follows:

(2) $$K_{i\pm c}=\{\begin{array}{l} \left(x_{i+c},y_{i+c}\right)=\left(x_{i}+c,y_{i}+\sum_{i}^{i+c}∆y\right)\\ \left(x_{i-c},y_{i-c}\right)=\left(x_{i}-c,y_{i}+\sum_{i-c}^{i}∆y\right) \end{array}$$

In the *y*-axis direction:

(3) $$K_{j\pm c}=\{\begin{array}{l} \left(x_{j+c},y_{j+c}\right)=\left(x_{j}+\sum_{j}^{j+c}∆x,y_{j}+c\right)\\ \left(x_{j-c},y_{j-c}\right)=\left(x_{j}+\sum_{j-c}^{j}∆x,y_{j}-c\right) \end{array}$$

As shown in Figure 8b, during the deformation process, DSConv covers a range of 9 × 9, expanding the model’s receptive field. This adaptation better accommodates the dynamic changes in beef cattle posture, enabling the perception of key behavioral features for accurate identification and tracking of beef cattle behaviors, laying a solid foundation for the process.

##### Introduction to the BiFormer Module

In real beef cattle farming scenarios, which are inherently complex, to establish better connectivity between global and local features of beef cattle behavior in images, focusing on key features, this paper introduces a lightweight and efficient BiFormer [23] attention mechanism. The BiFormer attention mechanism exhibits dynamic sparsity and efficient computation, reducing the loss of beef cattle behavior features in complex scenarios characterized by challenging lighting conditions and severe occlusions, thereby enhancing the performance of the target network.

**Figure 9:**
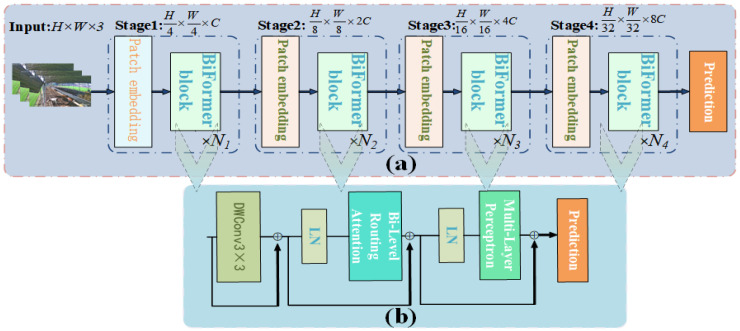
BiFormer flowchart. (**a**) Overall structure. (**b**) Detailed structure of BiFormer module.

The dual-level routing attention (BRA) in the BiFormer module filters out most irrelevant key–value pairs in coarse-grained regions, retaining a small number of relevant routing regions and utilizing fine-grained attention mechanisms to focus on beef cattle behavior regions at the pixel level, embodying dynamic sparsity characteristics. The overall structure of the BiFormer module, as shown in Figure 9a, involves initial image input, followed by embedding overlapping blocks in the first stage, optimization of the module in stages two to four, doubling the input spatial resolution and channel numbers, and transforming beef cattle behavior features through Ni consecutive BiFormer blocks. Within the BiFormer block, a 3 × 3 depth convolution implicitly encodes relative positional information, while the dual-level routing attention (BRA) module and multi-layer perceptron (MLP) module, respectively, handle the modeling of cross-position relationships and embedding of all positions, as depicted in Figure 9b.

#### 2.2.2. Re-Identification Network Improvement

**Figure 10:**
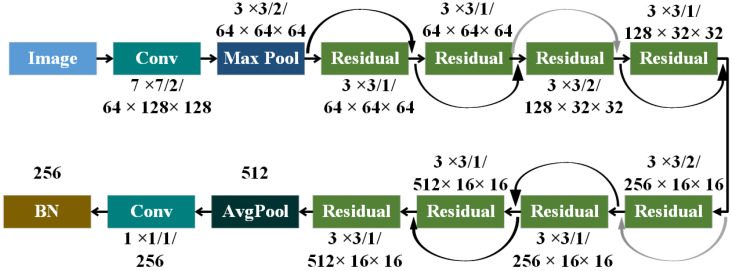
ResNet18 structure diagram.

The accuracy of tracking is directly proportional to the precision of appearance feature information; in other words, the more accurate the appearance feature information, the higher the tracking accuracy. Therefore, this paper replaces the original network structure with the ResNet18 structure, which provides better convolutional effects and higher-dimensional vector features. The structure of ResNet18 is represented in the following Figure 10 and Table 2.

It includes 2 convolutional layers, 1 max pooling layer, and an average pooling layer, as well as 8 residual modules with L2 normalization to map features onto a unit hypersphere, reducing computational load to further utilize cosine similarity for measuring similarity.

To obtain more feature information from the feature maps, this paper sets the stride of Residual 9 to 1, thus enhancing the resolution of the feature map and capturing additional feature information. Furthermore, to enhance feature matching, this paper introduces triplet loss after the 256-dimensional feature space, ensuring the discriminative ability of the 256-dimensional output features. By constraining the network parameters through the stride, this paper achieves higher feature matching. Triplet loss [24] is a mathematical function mapping in computational mathematics. In the process of feature extraction, the matching degree between two vectors is measured using the distance Formula (4). A smaller distance indicates a smaller difference between the two vector features, while a larger distance indicates a larger difference.

(4) $$d_{\mathrm{cosine}}(A,B)=1-\frac{A\cdot B}{\left|A\right|\left|B\right|}$$

#### 2.2.3. Improvement in Trajectory Generation and Matching Process

In experiments involving multi-target tracking of group-raised beef cattle, discrepancies between trajectory prediction boxes and detection boxes arise due to instances wherein target beef cattle are obscured or disappear for extended periods during their movement, resulting in the creation of new trajectories. Consequently, as video frames progress, the same beef cattle are assigned different IDs at different moments, leading to a significant mismatch between the maximum ID value and the actual number of beef cattle. To mitigate the impact of these issues, this paper improved the algorithm’s trajectory generation and matching processes.

**Figure 11:**
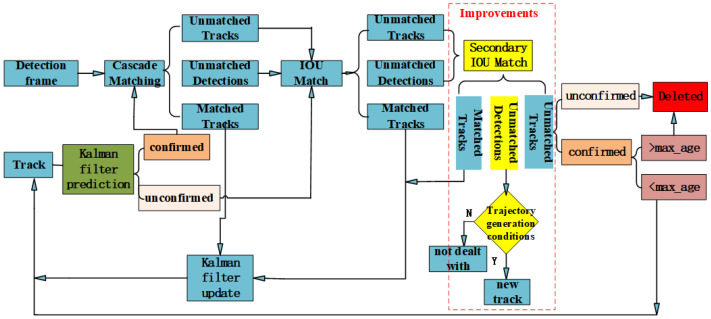
Trajectory generation and matching process.

In real-world environments wherein beef cattle are group-raised, the cattle pens are enclosed, and the number of beef cattle is fixed. Additionally, the maximum ID value representing the number of beef cattle remains constant. To enhance matching accuracy and reduce instances of mismatch due to excessively large IDs, secondary IOU matching was introduced, with a relaxation in the distance criteria for this matching. This adjustment significantly reduces cases of trajectory mismatches and detection box mismatches in the initial IOU matching process, as illustrated in Figure 11.

Due to the inherent presence of false positives and false negatives in tracking algorithms, the maximum ID value in this paper is dynamically adjusted and referred to as the extreme ID value. The extreme ID value is determined by the average of the maximum IDs from the last 5 frames. If the average value is an integer, no adjustments are made; however, if there is a decimal fraction, it is rounded up to the nearest whole number.

In Figure 11, the condition for trajectory generation is as follows: the current trajectory’s ID must be less than the extreme ID value. If this condition holds true, indicating that the current trajectory’s ID does not exceed the extreme ID value, a new trajectory is generated. Conversely, if the current trajectory’s ID surpasses the extreme ID value due to false positives or false negatives, no new trajectory is created.

The trajectory generation process in Figure 11 differs from that of Deep SORT primarily in the improved sections; the rest remains the same.

## 3. Results and Analysis

### 3.1. Experimental Environment

The experimental setup in this paper maintains identical hardware and software configurations. It utilizes an NVIDIA GeForce RTX 4070 GPU (NVIDIA, Santa Clara, CA, USA) with 16 GB of memory and an Intel Core i7-13700F 3.0GHz (Intel (China), Minhang District, Shanghai, China)processor with 16GB of RAM. The experiments are conducted in the PyTorch 2.0.0 framework, under the environment of CUDA 11.8 and CuDNN 8700.

For training the beef cattle behavior identification algorithm, the training runs for 200 epochs with a batch size of 32. The network parameters are optimized using the SGD optimizer with an initial learning rate of 0.002, a momentum of 0.94, and a weight decay coefficient set to 0.0015. The learning rate is adjusted using the cosine annealing algorithm.

In the training of the re-identification model, the training runs for 100 epochs with a batch size of 32. The momentum is set to 0.92, and the optimizer is SGD with an initial learning rate of 0.001 and a weight decay coefficient of 0.000001.

### 3.2. Model Evaluation

#### Evaluation Metrics for Beef Cattle Behavior Detection Algorithm

The evaluation metrics for the beef cattle behavior detection algorithm are as follows: P (precision) represents the proportion of correctly predicted positive beef cattle behaviors, i.e., accuracy; R (recall) represents the proportion of actual positive beef cattle behaviors correctly predicted as positive, i.e., recall; AP (average precision) represents the average accuracy; mAP (mean average precision) represents the average of AP values across all classes. When considering IOU thresholds of 0.5 and 0.5 to 0.95, they are denoted as mAP50 and mAP50:95, respectively.

(5) $$Precision=\frac{TP^{1}}{TP+FP^{2}}$$

(6) $$Recall=\frac{TP}{TP+FN^{3}}$$

(7) $$AP=\int_{0}^{1}P(R)dR$$

^1^ The number of correctly detected beef cattle behaviors. ^2^ The number of falsely detected beef cattle behaviors. ^3^ The number of missed beef cattle behaviors.

This paper mainly uses the following four evaluation metrics for target tracking: ID switching count (ID_switch, IDS) is the total number of individual ID tracking switches in beef cattle; multiple object tracking precision (MOTP), measures the mismatch between annotated and predicted bounding boxes; multiple object tracking accuracy (MOTA) measures the extent of false alarms, missed targets, and identity switches; identification F1 (IDF1) is a metric that compares the correct detections to the average true number and the computed detection count.

The calculation formula for MOTA is shown in Equation (8).

(8) $$MOTA=1-\frac{\left(FN+FP+IDS\right)}{GT^{4}}\in (-\infty ,1]$$

^4^ The total number of ground truth-bounding boxes in the video.

The specific expression for MOTP is given by Equation (9). In the equation, ‘$c_{t}$’ represents the number of successful matches in frame *t*, and ‘$d_{i,t}$’ is the intersection over union (IoU) between the successfully matched ground truth and predicted bounding boxes in frame t.

(9) $$MOTP=\frac{\sum_{i,t}d_{t}^{i}}{\sum_{t}c_{t}}$$

The calculation formula for IDF1 is depicted in Equation (10).

(10) $$IDF1=\frac{2TP}{2TP+FP+FN}$$

### 3.3. Results of the Beef Cattle Behavior Detection Algorithm

#### 3.3.1. Ablation Experiment

To further illustrate the impact of different improvement modules on beef cattle posture identification, this paper conducted the ablation experiments shown in Table 3. The experiments were carried out using the method of controlled variables, where all aspects such as hardware, software, and datasets remained consistent except for the varied modules (“×” and “√” in the table represent not adopted and adopted for the respective modules). The results of the experiments indicate that the algorithm incorporating the Dynamic Snake Convolution (DSConv) and BiFormer attention mechanism showed varying degrees of improvement in evaluation metrics compared to the original YOLOv8n. This demonstrates the feasibility and optimization effects of different improvement modules on beef cattle posture identification. The introduction of different improvement points had varying effects, with the algorithm YOLOv8n_BiF_DSC incorporating both improvement points and outperforming YOLOv8n_DSC and YOLOv8n_BiF, which each introduced a single improvement point, achieving higher levels across all evaluation metrics, with an impressive increase of up to 7.1% in the average precision of 50:95.

YOLOv8n_BiF_DSC exhibited continued effectiveness in identifying behaviors even with larger IOU thresholds, showcasing high robustness. Although ranking slightly lower in recall rate at 92.9%, YOLOv8n_BiF_DSC remains a high-recall algorithm. Overall, YOLOv8n_BiF_DSC excelled in accuracy, average precision 50, and 50:95 compared to the original YOLOv8n, with increments of 5.3%, 5.2%, and 7.1%, respectively. This highlights the practicality and effectiveness of the YOLOv8n_BiF_DSC algorithm in beef cattle behavior identification.

**Figure 12:**
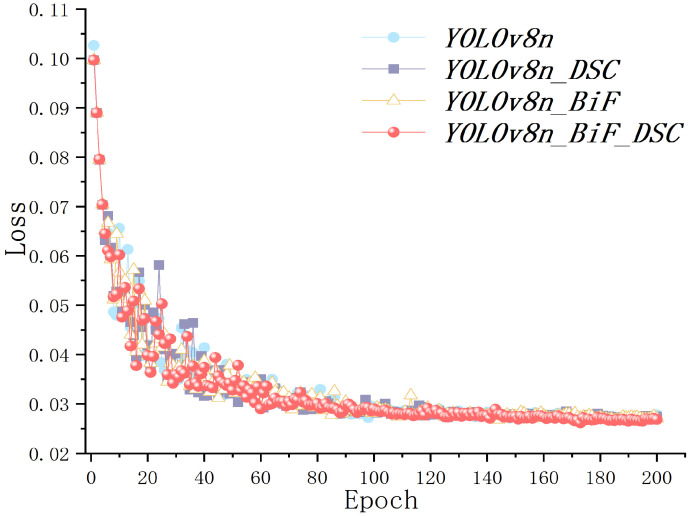
Loss curve.

The loss values of each algorithm in the ablation experiments are depicted in Figure 12. The horizontal axis represents the number of algorithm iterations, while the vertical axis represents the degree of inconsistency between the predicted and actual values at each iteration. A lower loss value indicates higher algorithm robustness. From the graph, it can be observed that the loss values of each algorithm first rapidly converge, then exhibit slight oscillations, and finally converge completely around 0.025–0.03. The training process did not show signs of underfitting or overfitting, indicating the usability and effectiveness of the algorithm.

#### 3.3.2. The Detection Results and Visual Analysis of the YOLOv8n_BiF_DSC

**Figure 13:**
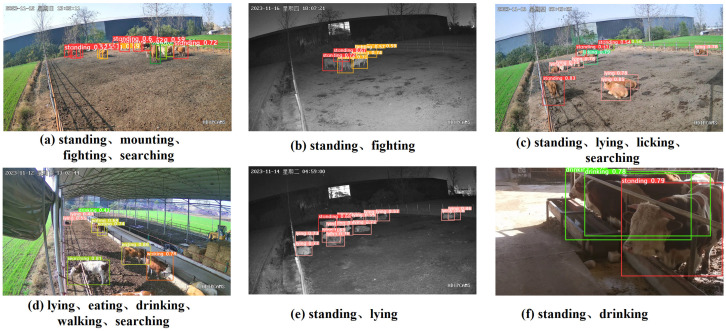
Beef cattle behavioral detection chart.

The YOLOv8n_BiF_DSC algorithm for identifying nine different behaviors of beef cattle is presented in Figure 13 of this paper.

From the above figure, it can be observed that Figure 13a–e shows the results of beef cattle behavior identification at different times using the stationary YOLOv8n_BiF_DSC algorithm. Figure 13a,c,d represents daytime identification images, while Figure 13b,e depicts nighttime identification images. Except for a small portion of the beef cattle behaviors in Figure 13d that were not detected due to their extreme distance, the standing, mounting, fighting, searching, lying, eating, drinking, working, and licking behaviors were successfully detected in the other images. Figure 13f displays the results of identification from mobile filming, with the standing and drinking behaviors of the cattle being identified. In conclusion, the YOLOv8n_BiF_DSC algorithm demonstrates high feasibility in identifying multiple behaviors of beef cattle in various farming scenarios.

This paper introduces Grad-CAM++ [25] for a better explanation of the model’s prediction effects, as shown in the following figure. The higher the attention, the redder the target region, and the lower the attention, the bluer the region.

**Figure 14:**
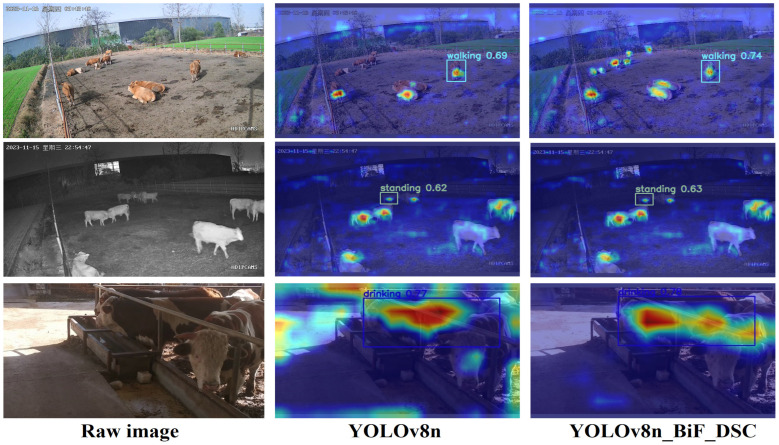
Visualization map.

As depicted in Figure 14, the YOLOv8n_BiF_DSC model used in this study focuses more on beef cattle behaviors. Despite some background noise interference, the key features of beef cattle behaviors were successfully extracted, showing a significant improvement over the original YOLOv8n model. This further emphasizes the feasibility of YOLOv8n_BiF_DSC in practical beef cattle farming.

#### 3.3.3. The YOLOv8n_BiF_DSC Algorithm Compared with Other Models

To further demonstrate the feasibility of the YOLOv8n_BiF_DSC algorithm, this study conducted comparative experiments with the YOLOv5, YOLOv7, YOLOv9 [26], and YOLOv10 [27] algorithms. The experimental results are presented in Table 4. The results indicate that the proposed YOLOv8n_BiF_DSC algorithm shows varying degrees of improvement in beef cattle pose recognition compared to the YOLOv5, YOLOv7, YOLOv9, and YOLOv10 algorithms. The proposed algorithm demonstrates notable enhancements over the best-performing YOLOv10 algorithm in terms of accuracy, recall rate, and average precision, with a significant increase of 5.3% in precision (P) and 5.5% in mean average precision 50:95 (mAP50:95). These results suggest that the YOLOv8n_BiF_DSC algorithm exhibits high feasibility in beef cattle behavior detection.

### 3.4. Re-Identification Network Model Loss and mAP Curve Analysis

**Figure 15:**
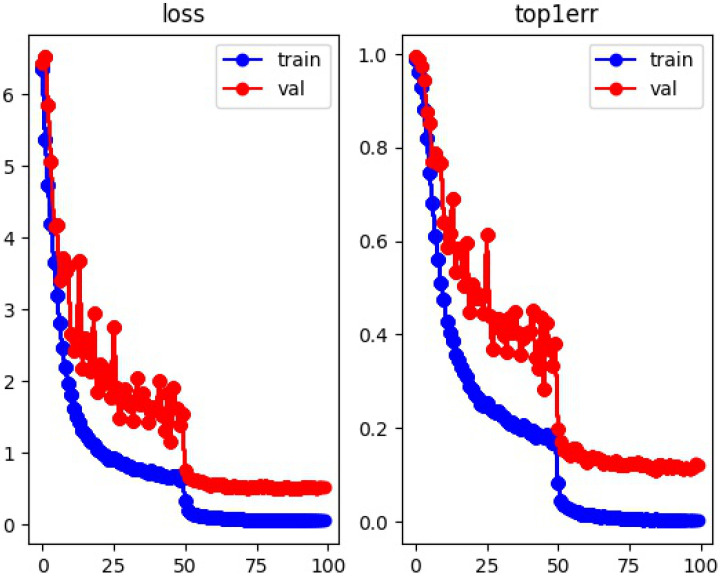
Convergence of loss value and top-1 error.

**Figure 16:**
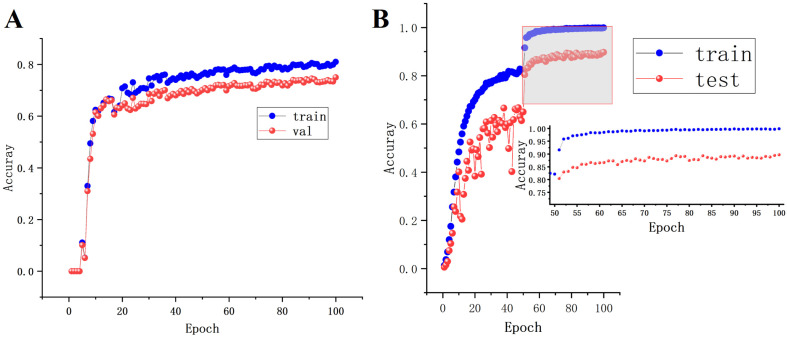
Accuracy curve graphs ((**A**) accuracy curve of the original algorithm; (**B**) accuracy curve of ResNet18).

The improved appearance feature re-identification network was iterated 100 times. The convergence graphs of the loss, top-1 error, and accuracy on the validation and training sets for this network are shown in Figure 15 and Figure 16, respectively. Figure 15 displays the loss curve on the left and the top-1 error curve on the right. The top-1 error represents the probability of misclassifying the highest probability class.

In the loss curve and top-1 error curve on the left and right sides of Figure 15, the horizontal axis represents the training epochs, while the vertical axis represents the values of the loss and classification error rate. The loss value and error rate decrease rapidly from Epoch 0 to 20. From Epoch 20 to 50, the decrease in the loss and error rate values for both the training and validation sets becomes more gradual. There are occasional minor oscillations in the validation set data during this period, but they can generally be disregarded. Finally, from Epoch 50 to 99, the loss and error rate values stabilize, with the loss values for the training and validation sets reaching around 0.001 and 0.5, respectively, and the error rate values approaching 0.001 and 0.11.

Figure 16A represents the accuracy curve of the original algorithm, while in Figure 16B, the left side depicts the accuracy curve of ResNet18, and the right side shows an enlarged view of the accuracy curve (Epochs 50–100). The horizontal axis represents the number of training iterations, and the vertical axis represents accuracy. From Figure 16, it is evident that the accuracy of both the original algorithm and ResNet18 gradually increases and stabilizes as epochs increase. Specifically, the accuracy of ResNet18 on the training and validation sets stabilizes around 0.99 and 0.89, respectively, significantly higher than the original accuracy of around 0.8 and 0.78. During the model training process, there were no occurrences of underfitting; the model converged and the weights were saved.

### 3.5. Analysis of Beef Cattle Multi-Object Tracking Results

#### 3.5.1. Tracking Experiment Results Comparison

To validate the performance of the beef cattle multi-object identification algorithm used in this paper, which involves changing detectors, re-identification networks, and improving the trajectory generation and matching processes, experiments were conducted under the condition of group-raised beef cattle. The results are shown in Table 5. The experiments were carried out using the selected video sequences 01–05, which mainly include nine behavioral characteristics of beef cattle: standing, lying, mounting, fighting, licking, eating, drinking, working, and searching.

According to Table 5, the improved IDF1 of the Deep SORT algorithm is 0.811, which is 4.1% higher than the original Deep SORT algorithm’s 0.77. This indicates that the improved Deep SORT algorithm can enhance tracking accuracy in various finishing environments.

In terms of IDS, the improved Deep SORT algorithm has an IDS of 40, which is 65.8% lower than the original Deep SORT algorithm’s IDS of 117. In the less active beef cattle video sequence 03, the IDS of the improved Deep SORT algorithm decreased from 4 to 0. Due to enhancements in trajectory generation and matching processes, the IDS of the improved Deep SORT algorithm in the highly active video sequence 05 decreased by 79% compared to the original Deep SORT algorithm. This suggests that the improved Deep SORT algorithm enhances beef cattle multi-object tracking in both simple and complex environments.

Regarding MOTA, the improved Deep SORT algorithm has a value of 0.921, a 2% increase over the original Deep SORT algorithm’s 0.901. In video sequences 01–05, the improved Deep SORT algorithm outperforms the original algorithm to varying degrees, indicating improved accuracy in beef cattle multi-object tracking across different environments. There is also a slight improvement in MOTP with the improved Deep SORT algorithm compared to the original.

In conclusion, the improved Deep SORT algorithm used in this study enhances the stability and accuracy of beef cattle multi-object tracking in group-raised cattle environments, making it suitable for intelligent beef cattle farming practices.

**Figure 17:**
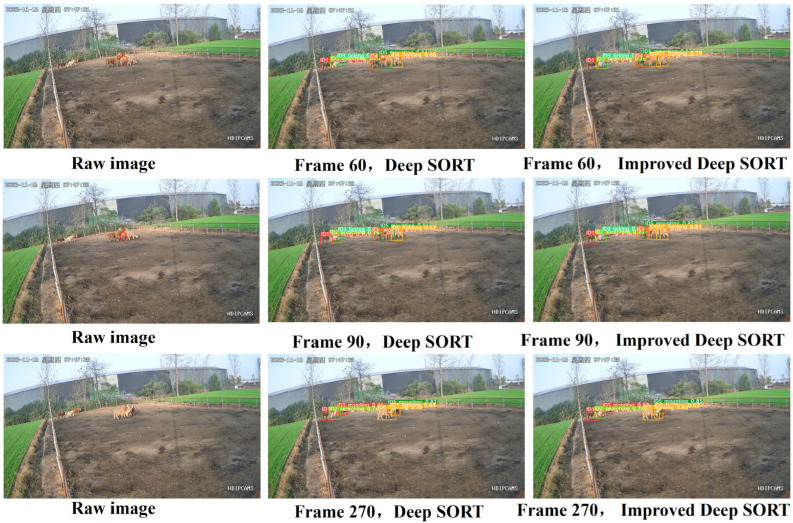
Track results before and after improvements. (“星期四” is Thursday).

By selecting generally active video sequences, visualizations of the tracking results of the improved Deep SORT algorithm and the original Deep SORT algorithm are presented in Figure 17 (I have marked the missed beef cattle with red circles).

As shown in the figure below, the improved Deep SORT algorithm can track all cattle in frames 60 and 90, even when severe occlusion is present. By frame 270, both the original and improved algorithms can track all cattle, but the fitting of the tracking boxes is notably better with the improved algorithm.

In conclusion, this study proposes that the improved tracking model based on the Deep SORT algorithm can provide technical support for the multi-object tracking of beef cattle in group-raised environments, contributing to the goal of achieving intelligent management of beef cattle.

#### 3.5.2. Sparse Cattle Group Algorithm Tracking Performance Analysis

**Figure 18:**
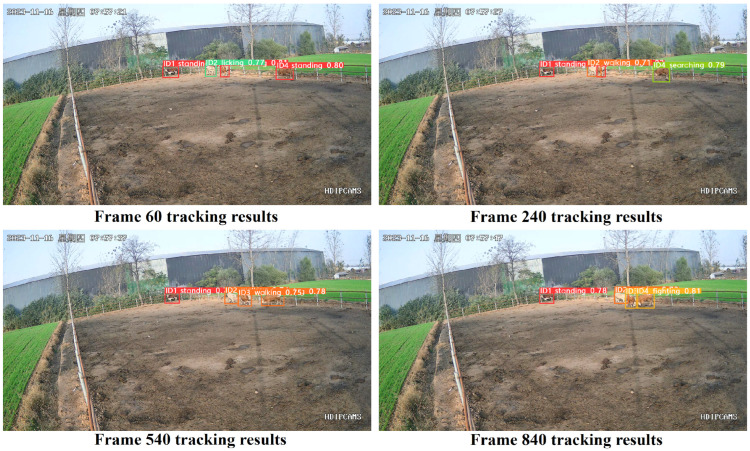
Sparse cattle herd tracking results. (“星期四” is Thursday).

The sparse cattle group video sequences consist of four beef cattle with the following behaviors: ID1 is always standing; ID2 includes licking and walking; ID3 involves standing, walking, and fighting; and ID4 includes standing, searching, walking, and fighting. Most of the time, the cattle are not occluded, with only brief occlusions occurring during fighting, as shown in Figure 18.

In the sparse cattle group scenario, there are no occlusion issues at frames 60 and 540, and the occlusion of cattle is minimal at frames 240 and 840 in the video. The trajectory generation and matching processes of the original Deep SORT algorithm were optimized. Consequently, the IDS significantly decreased with the improved Deep SORT algorithm, reaching an ID of zero during certain frames in the graph. Replacing the detector and re-identification network of the original Deep SORT algorithm can enhance detection accuracy and tracking precision. However, there are instances of inaccurate fitting of tracking boxes, particularly during changes in lighting conditions and rapid cattle movements, as seen in frame 540 of the video. Nevertheless, the improved Deep SORT algorithm can still outline the tracking boxes and maintain IDs, minimizing the impact on the multi-object tracking of beef cattle.

In summary, the improved Deep SORT algorithm performs well in tracking multi-objects in sparse cattle groups, reducing ID jumps compared to the original algorithm and enhancing tracking performance.

#### 3.5.3. Analysis of the Tracking Performance of Algorithms for Distant and Dense Cattle Groups

The density of beef cattle in this study is assessed through observations conducted by skilled livestock caretakers. Specifically, the criteria are as follows: An environment is deemed dense if there are three or more beef cattle per square meter; otherwise, it is considered non-dense. The video sequences of distant and dense cattle groups consist of seven beef cattle filmed from a distance. Among them, cattle with IDs 2 and 3 exhibit dense activities, severe occlusions, and brief losses during movement.

**Figure 19:**
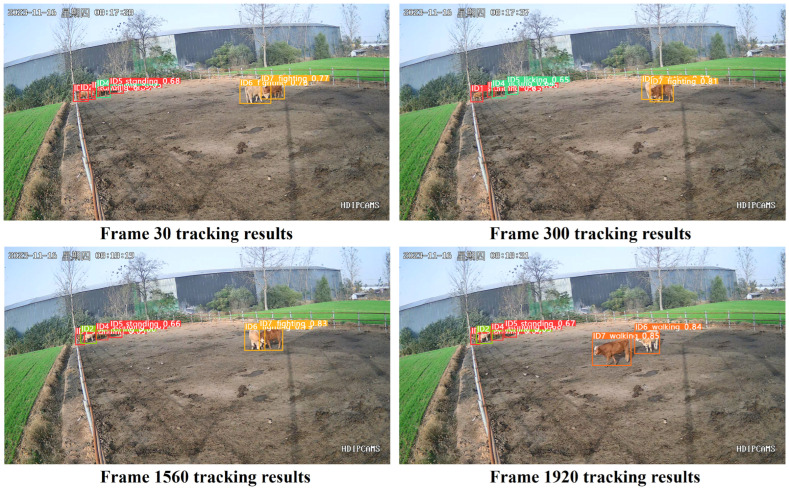
Remote dense cattle herd tracking results. (“星期四” is Thursday).

As shown in Figure 19, the improved Deep SORT algorithm successfully tracks cattle with IDs 1, 4, 5, 6, and 7 at frames 30, 300, 1560, and 1920, even when dealing with densely active cattle like IDs 6 and 7 with occlusions, without any ID jumps. At frame 300, due to severe occlusion, ID 2 briefly loses tracking, but it regains tracking when occlusion decreases at frame 1560 without any ID jumps. This improvement is attributed to the replacement of the detector in the improved Deep SORT algorithm, which enhances detection accuracy, and the replacement of the re-identification network, which improves the algorithm’s identification of appearance features. Additionally, optimizations in trajectory generation and matching processes reduce instances of trajectory mismatches and detection box misalignments, thereby enhancing the tracking performance of multi-object tracking of beef cattle. This lays a solid foundation for the intelligent farming of beef cattle.

#### 3.5.4. Comparative Analysis with Other Tracking Models

To further demonstrate the feasibility of the improved Deep SORT algorithm, this study conducted comparative experiments with the ByteTrack [28] and StrongSORT [29] algorithms. The experimental results are presented in Table 6. The results indicate that the proposed algorithm shows varying degrees of improvement in multi-target beef cattle tracking compared to the ByteTrack and StrongSORT algorithms. The proposed algorithm demonstrates notable enhancements over the best-performing StrongSORT algorithm in terms of IDF1, IDS, MOTA, and MOTP, with a significant increase of 1.7% in IDF1 (P) and 1% in MOTA, while IDS decreased by 42.9%. These results suggest that the improved Deep SORT algorithm exhibits high feasibility in multi-target beef cattle tracking.

## 4. Discussion

To improve the efficiency of beef cattle behavior identification and tracking, an increasing number of cattle practitioners are beginning to experiment with the non-invasive identification and tracking of beef cattle behaviors using deep learning models. Compared to traditional methods, this approach effectively reduces labor costs and enhances work efficiency, providing a new direction for the intelligent finishing and management of beef cattle.

Wang M et al. [30] proposed an optimized deep learning algorithm based on FairMOT, which improved features for re-identification and IOU matching modules. This algorithm achieved individual livestock identification and tracking in two sets of test videos, increasing the tracking accuracy of multiple livestock to 81.17% and 91.41%, respectively. Cowton J. et al. [31] introduced a combination of region-based convolutional neural networks and real-time multi-object tracking methods to create a system for detecting and tracking multiple livestock. This system autonomously locates and tracks individual livestock, achieving a target tracking accuracy of 92%.

The experiments in this study show that: (1) In terms of beef cattle behavior identification, the YOLOv8n_BiF_DSC algorithm achieves an average precision of 96.5% in identifying nine behaviors such as standing, lying, mounting, fighting, licking, eating, drinking, moving, and searching in various complex scenarios like different lighting conditions and densities. This partially addresses the challenges of low accuracy and difficulty in beef cattle behavior identification. (2) Regarding beef cattle tracking, experiments at five different complexity levels of test video sequences demonstrate that the improved Deep SORT algorithm shows varying degrees of improvement in IDF1, IDS, MOTA, and MOTP compared to the original tracking algorithm. The IDS is reduced by 65.8%, and the MOTA is increased by 2%. This enhancement addresses issues of missed detections and false alarms in tracking group-raised beef cattle in sparse and distantly dense environments, thus facilitating better tracking of beef cattle and laying the groundwork for intelligent detection and tracking in beef cattle farming. In the future, by integrating with online platforms for beef cattle behavior identification and tracking, practitioners can observe beef cattle around the clock, facilitating targeted statistical and predictive analyses of essential livestock such as calves and cows at critical time points.

Due to limitations in experimental time, space, budget, and related hardware, there is still room for improvement in data collection and the hardware used in the experiments, which could lead to obtaining clearer and more precise data images and videos. Additionally, as hardware performance improves, the runtime of algorithms will significantly decrease. While this study focuses on beef cattle, the algorithm proposed herein remains highly feasible and valuable for application to other livestock species.

## 5. Conclusions

This study focuses on group-raised beef cattle in finishing farms, collecting data on group-raised beef cattle using cameras and related hardware devices. Addressing issues such as low production efficiency and poor livestock welfare in traditional finishing farms, a deep learning-based computer vision algorithm for intelligent detection and tracking of group-raised beef cattle was proposed. The specifics are as follows:

(1) The beef cattle behavior detection module, based on the YOLOv8n algorithm, first introduces the Dynamic Snake Convolution module to enhance the capability of extracting key features of beef cattle behaviors and expand the model’s receptive field. Subsequently, the BiFormer attention mechanism is introduced to integrate high-level and low-level feature information, dynamically and sparsely learning beef cattle behavior features. The improved YOLOv8n_BiF_DSC algorithm achieves identification accuracy rates of 93.6% for nine beef cattle behaviors, with an average precision of 96.5%, and precision at 50:50 and 50:95 thresholds of 71.5% and 96.5%, respectively, representing increases of 5.3%, 5.2%, and 7.1% compared to the original YOLOv8n.
(2) The beef cattle multi-target tracking module, based on the Deep SORT algorithm, first replaces the detector with YOLOv8n_BiF_DSC to improve the detector’s detection accuracy. Subsequently, the re-identification network model is replaced to enhance the tracking algorithm’s ability to obtain appearance information. Finally, secondary IOU matching is added to optimize the trajectory generation and matching processes of the Deep SORT algorithm, reducing instances of ID mismatches and misalignments in tracking. Experiments conducted on five test video sequences of varying complexity levels show that the improved Deep SORT algorithm exhibits improvements in IDF1, IDS, MOTA, and MOTP compared to the original tracking algorithm, with IDS reduced by 65.8% and MOTA increased by 2%. This enhancement addresses issues of missed detections and false alarms in tracking group-raised beef cattle in sparse and dense environments, thus facilitating the better tracking of beef cattle and laying the groundwork for intelligent detection and tracking in beef cattle farming.

Future efforts will focus on expanding the dataset to cover a wider range of scenarios, improving hardware capabilities, and further validating the algorithm’s feasibility and robustness to provide data and technological support for intelligent beef cattle farming and management.

## Figures and Tables

**Table 1:** Beef cattle behavior determination rules and example information.

Behavior Number	Behavioral Categories/Labels	Behavioral Description	Instances
(1)	standing	Standing supported by 4 cow legs, cow head not touching the ground	10,422
(2)	lying	Legs bent and touching the ground or body touching the ground	10,800
(3)	mounting	A cow’s front hooves crawling across another cow’s body	1518
(4)	fighting	Two or more bulls headbutting each other	5800
(5)	licking	Licking the face and torso with the tongue	1600
(6)	eating	Cow’s head enters the trough	6000
(7)	drinking	Cow’s head into the water trough	1680
(8)	walking	Four cow legs displaced from the carcass, cow head not touching the ground	5400
(9)	searching	Cow’s head near or touching the ground	10,300

**Table 2:** Structure of Resnet18.

Network Structure Name	Core Size	Step Size	Output Feature Map Size
Conv1 + BN + Relu	7 × 7	2	64 × 128 × 128
Max Pool 2	3 × 3	2	64 × 64 × 64
Residual 3	3 × 3	1	64 × 64 × 64
Residual 4	3 × 3	1	64 × 64 × 64
Residual 5	3 × 3	2	128 × 32 × 32
Residual 6	3 × 3	1	128 × 32 × 32
Residual 7	3 × 3	2	256 × 16 × 16
Residual 8	3 × 3	1	256 × 16 × 16
Residual 9	3 × 3	1	512 × 16 × 16
Residual 10	3 × 3	1	512 × 16 × 16
AdaptiveAvgPool 11	-	-	512
Conv 12	1 × 1	1	256
Batch and 12 normalization	-	-	256

**Table 3:** Ablation experiment identification results.

Model	DSC	BiF	P	R	mAP50	mAP50:95
YOLOv8n	×	×	0.883	0.866	0.913	0.644
YOLOv8n_DSC	√	×	0.933	0.894	0.945	0.677
YOLOv8n_BiF	×	√	0.922	0.931	0.94	0.672
YOLOv8n_BiF_DSC	√	√	0.936	0.929	0.965	0.715

**Table 4:** Other model identification results.

Model	P	R	mAP50	mAP50:95
YOLOv5	0.861	0.853	0.889	0.625
YOLOv7	0.87	0.862	0.911	0.642
YOLOv9	0.881	0.871	0.913	0.643
YOLOv10	0.883	0.884	0.92	0.66
YOLOv8n_BiF_DSC	0.936	0.929	0.965	0.715

**Table 5:** Tracking algorithm performance comparison.

Model	Video Sequence	IDF1	IDS	MOTA	MOTP
Deep SORT	01	0.787	25	0.879	0.856
	02	0.824	15	0.907	0.868
	03	0.861	4	0.925	0.864
	04	0.829	11	0.909	0.86
	05	0.551	62	0.883	0.867
	Total/Average	0.77	117	0.901	0.863
Improved Deep SORT (ours)	01	0.791	12	0.905	0.86
	02	0.843	8	0.92	0.884
	03	0.899	0	0.948	0.875
	04	0.89	7	0.93	0.865
	05	0.632	13	0.901	0.871
	Total/Average	0.811	40	0.921	0.871

**Table 6:** Results of other tracking models’ recognition.

Model	IDF1	IDS	MOTA	MOTP
Deep SORT	0.77	117	0.901	0.863
ByteTrack	0.79	80	0.907	0.864
StrongSORT	0.794	70	0.911	0.869
Improved Deep SORT (ours)	0.811	40	0.921	0.871

## Data Availability

The data presented in this study are available upon request from the corresponding author. The data are not publicly available due to privacy and confidentiality.

## References

[1] Li G., Erickson G.E., Xiong Y. (2022). Individual beef cattle identification using muzzle images and deep learning techniques. Animals.

[2] Estrada O.R., Almeida F.R., Utsumi S., Fredrickson E., Enríquez G.B., Cibils A., Estell R., Gonzalez A. (2023). Foraging behavior of Raramuri Criollo vs. commercial crossbred cows on rangelands of the southwestern United States and Northern Mexico. J. Arid Environ..

[3] Li G., Shi G., Zhu C. (2024). Dynamic Serpentine Convolution with Attention Mechanism Enhancement for Beef Cattle Behavior Recognition. Animals.

[4] Song C., Jiang D., Wang F. (2023). Exploration of agricultural IoT breeding tracking based on a saliency visual target tracking algorithm. Turk. J. Agric. For..

[5] Nyamuryekung’E S., Duff G.C., Estell R., Utsumi S., Funk M., Cibils A., Cox A., Gong Q., Cao H., Spiegal S. (2022). PSXIII-7 Field Testing of Lora-wan Sensors for Real-Time Tracking and Biosensing of Brangus and Raramuri Criollo Cattle Grazing a Small Pasture. J. Anim. Sci..

[6] Gwatirisa C., Mudereri B., Chitata T., Mukanga C., Ngwenya M., Muzvondiwa J., Mugandani R., Sungirai M. (2022). Microhabitat and patch selection detection from GPS tracking collars of semi-free ranging Mashona cattle within a semi-arid environment. Livest. Sci..

[7] Zheng Z., Qin L. (2023). PrunedYOLO-Tracker: An efficient multi-cows basic behavior recognition and tracking technique. Comput. Electron. Agric..

[8] Zheng Z., Li J., Qin L. (2023). YOLO-BYTE: An efficient multi-object tracking algorithm for automatic monitoring of dairy cows. Comput. Electron. Agric..

[9] Bhujel A., Arulmozhi E., Moon B.-E., Kim H.-T. (2021). Deep-Learning-Based Automatic Monitoring of Pigs’ Physico-Temporal Activities at Different Greenhouse Gas Concentrations. Animals.

[10] Tassinari P., Bovo M., Benni S., Franzoni S., Poggi M., Mammi L.M.E., Mattoccia S., Di Stefano L., Bonora F., Barbaresi A. (2021). A computer vision approach based on deep learning for the detection of dairy cows in free stall barn. Comput. Electron. Agric..

[11] Myat Noe S., Zin T.T., Tin P., Kobayashi I. (2023). Comparing state-of-the-art deep learning algorithms for the automated detection and tracking of black cattle. Sensors.

[12] Psota E.T., Schmidt T., Mote B., CPérez L. (2020). Long-term tracking of group-housed livestock using keypoint detection and map estimation for individual animal identification. Sensors.

[13] Brunet D., Vrscay E.R., Wang Z. (2011). On the mathematical properties of the structural similarity index. IEEE Trans. Image Process..

[14] Wojke N., Bewley A., Paulus D. Simple online and realtime tracking with a deep association metric. Proceedings of the 2017 IEEE International Conference on Image Processing (ICIP).

[15] Bewley A., Ge Z., Ott L., Ramos F., Upcroft B. Simple online and realtime tracking. Proceedings of the 2016 IEEE International Conference on Image Processing (ICIP).

[16] Ren S., He K., Girshick R., Sun J. (2016). Faster R-CNN: Towards real-time object detection with region proposal networks. IEEE Trans. Pattern Anal. Mach. Intell..

[17] Redmon J., Divvala S., Girshick R., Farhadi A. You only look once: Unified, real-time object detection. Proceedings of the 2016 IEEE Conference on Computer Vision and Pattern Recognition (CVPR).

[18] Redmon J., Farhadi A. YOLO9000: Better, faster, stronger. Proceedings of the 2017 IEEE Conference on Computer Vision and Pattern Recognition (CVPR).

[19] Redmon J., Farhadi A. (2018). Yolov3: An incremental improvement. arXiv.

[20] Bochkovskiy A. (2020). Yolov4: Optimal speed and accuracy of object detection. arXiv.

[21] Wang C.Y., Bochkovskiy A., Liao H.Y.M. YOLOv7: Trainable bag-of-freebies sets new state-of-the-art for real-time object detectors. Proceedings of the IEEE/CVF Conference on Computer Vision and Pattern Recognition.

[22] Qi Y., He Y., Qi X., Zhang Y., Yang G. Dynamic snake convolution based on topological geometric constraints for tubular structure segmentation. Proceedings of the IEEE/CVF International Conference on Computer Vision.

[23] Zhu L., Wang X., Ke Z., Zhang W., Lau R.W. BiFormer: Vision Transformer with Bi-Level Routing Attention. Proceedings of the IEEE/CVF Conference on Computer Vision and Pattern Recognition.

[24] Fang Z., Ren J., Marshall S., Zhao H., Wang Z., Huang K., Xiao B. (2020). Triple loss for hard face detection. Neurocomputing.

[25] Chattopadhay A., Sarkar A., Howlader P., Balasubramanian V.N. Grad-cam++: Generalized gradient-based visual explanations for deep convolutional networks. Proceedings of the 2018 IEEE Winter Conference on Applications of Computer Vision (WACV).

[26] Wang C.Y., Yeh I.H., Liao H.Y.M. (2024). Yolov9: Learning what you want to learn using programmable gradient information. arXiv.

[27] Wang A., Chen H., Liu L., Chen K., Lin Z., Han J., Ding G. (2024). Yolov10: Real-time end-to-end object detection. arXiv.

[28] Zhang Y., Sun P., Jiang Y., Yu D., Weng F., Yuan Z., Luo P., Liu W., Wang X. (2022). Bytetrack: Multi-object tracking by associating every detection box. European Conference on Computer Vision.

[29] Du Y., Zhao Z., Song Y., Zhao Y., Su F., Gong T., Meng H. (2023). Strongsort: Make Deep SORT great again. IEEE Trans. Multimed..

[30] Wang M., Larsen M.L., Liu D., Winters J.F., Rault J.-L., Norton T. (2022). Towards re-identification for long-term tracking of group housed pigs. Biosyst. Eng..

[31] Cowton J., Kyriazakis I., Bacardit J. (2019). Automated individual pig localisation, tracking and behaviour metric extraction using deep learning. IEEE Access.

